# P-1711. Evaluation of Microbiology and Antibiotic Prescribing Behavior of Foot Puncture Wound Infection: An Opportunity for Antimicrobial Stewardship

**DOI:** 10.1093/ofid/ofae631.1877

**Published:** 2025-01-29

**Authors:** Brian Kim, Niki Arab, Arthur Jeng

**Affiliations:** Olive View-UCLA Medical Center, Sylmar, California; Olive View- UCLA Medical Center, Los Angeles, California; Olive View UCLA Medical Center/UCLA School of Medicine, Sylmar, California

## Abstract

**Background:**

The primary pathogen(s) of soft tissue infections, including those of the foot, are *β-hemolytic Streptococci* and *Staphylococcus aureus* (SA). *Pseudomonas aeruginosa* (PsA) has been implicated as a cause of foot infections after sustaining a nail puncture wound through a sneaker, historically leading to recommendations for prescribing empiric anti-PsA antibiotics (abx). At Olive View-UCLA Medical Center (Sylmar, CA), we evaluated the microbiology and empiric abx prescribing behavior of foot puncture wound infections.

**Figure:**
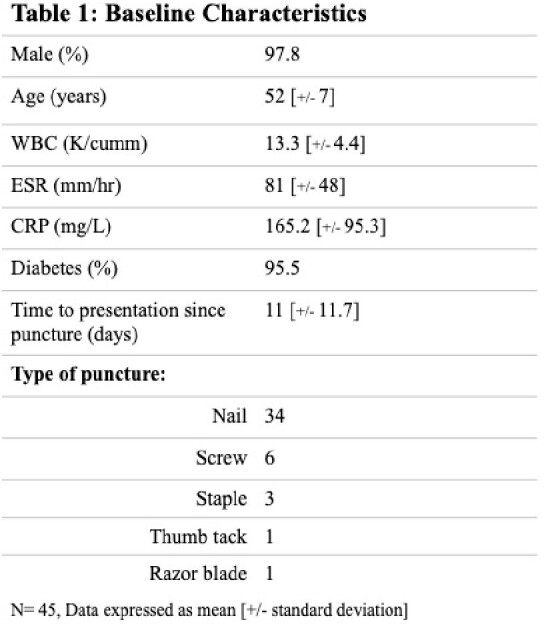

**Methods:**

Retrospective chart review on adults (≥ 18 years) admitted with an ICD-10 code for puncture wound from January 1, 2017 to December 31, 2023. Inclusion criteria included foot infection after sustaining nail, screw, staple, thumbtack, or razorblade puncture. Non-foot puncture wound and puncture from an object other than those listed in the inclusion criteria were excluded. Anti-PsA abx was defined as either piperacillin-tazobactam, cefepime, ceftazidime, or ciprofloxacin.

**Figure:**
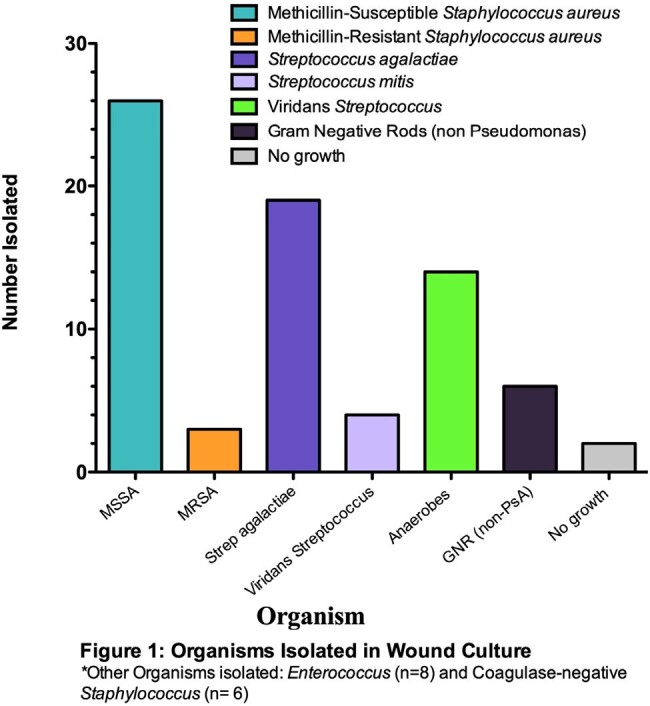

**Results:**

58 cases were reviewed and 45 met inclusion criteria. The majority of puncture wound was due to nail (n=34, 76%) followed by screw (n=6, 13%) [Table 1]. Empiric anti-PsA abx was prescribed in the emergency department in 41/45 (91%); empiric anti-PsA abx was prescribed or continued by the admitting physician in 19/45 (42%). Anti-PsA abx average days of therapy (DOT) was 2 days [IQR 2-3]. Wound culture was obtained in 44 cases, with no isolation of PsA. The majority of pathogens consisted of methicillin-susceptible SA (26/44 cases, 59%) and *Streptococcus agalactiae* (19/44, 43%) [Figure 1]. Infection involving both SA and S*treptococcus* spp. was seen in 15 cases (34%). Subsequent foot infection with PsA within 30 days of puncture wound infection did not occur in any cases.

**Conclusion:**

PsA was not isolated in any cases of foot puncture wound infections, although the majority were started on an empiric anti-PsA abx, per historical recommendations. The primary pathogen(s) were SA and/or *Streptococcus* spp. Therefore, current evidence shows that PsA is not a significant pathogen of foot puncture wound infections, and this represents an opportunity for antimicrobial stewardship services to prioritize empiric abx against SA and *Streptococcus* spp., and not against PsA, for this infection type.

**Disclosures:**

**All Authors**: No reported disclosures

